# Novel ways to explore surgical interventions in randomised controlled trials: applying case study methodology in the operating theatre

**DOI:** 10.1186/s13063-015-1127-x

**Published:** 2015-12-28

**Authors:** Natalie S. Blencowe, Jane M. Blazeby, Jenny L. Donovan, Nicola Mills

**Affiliations:** Centre for Surgical Research, School of Social and Community Medicine, University of Bristol, 39 Whatley Road, Bristol, BS8 2PS, UK; Division of Surgery, Head and Neck, University Hospitals Bristol NHS Foundation Trust, Upper Maudlin Street, Bristol, BS2 8HW, UK; National Institute for Health Research Collaboration for Leadership in Applied Health Research and Care West, University Hospitals Bristol NHS Foundation Trust, Upper Maudlin Street, Bristol, BS2 8HW, UK

**Keywords:** Randomised controlled trials, Qualitative research methods, Surgery, Intervention description and standardisation, Adherence

## Abstract

**Background:**

Multi-centre randomised controlled trials (RCTs) in surgery are challenging. It is particularly difficult to establish standards of surgery and ensure that interventions are delivered as intended. This study developed and tested methods for identifying the key components of surgical interventions and standardising interventions within RCTs.

Qualitative case studies of surgical interventions were undertaken within the internal pilot phase of a surgical RCT for obesity (the By-Band study). Each case study involved video data capture and non-participant observation of gastric bypass surgery in the operating theatre and interviews with surgeons. Methods were developed to transcribe and synchronise data from video recordings with observational data to identify key intervention components, which were then explored in the interviews with surgeons.

**Results:**

Eight qualitative case studies were undertaken. A novel combination of video data capture, observation and interview data identified variations in intervention delivery between surgeons and centres. Although surgeons agreed that the most critical intervention component was the size and shape of the gastric pouch, there was no consensus regarding other aspects of the procedure. They conceded that evidence about the ‘best way’ to perform bypass was lacking and, combined with the pragmatic nature of the By-Band study, agreed that strict standardisation of bypass might not be required.

**Conclusions:**

This study has developed and tested methods for understanding how surgical interventions are designed and delivered delivered in RCTs. Applying these methods more widely may help identify key components of interventions to be delivered by surgeons in trials, enabling monitoring of key components and adherence to the protocol. These methods are now being tested in the context of other surgical RCTs.

**Trial registration:**

Current Controlled Trials ISRCTN00786323, 05/09/2011.

## Background

There has been a lack of well-designed and conducted randomised controlled trials (RCTs) in surgery, partly because of methodological challenges [1]. One of these challenges relates to surgical interventions being considered as complex – i.e. containing multiple components that can be delivered in different ways by different surgeons [2–5]. This means that the ‘active ingredients’ (or ‘key components’) of surgical interventions may be uncertain and as such, deciding on the extent to which they should be described and standardised within trials can be challenging. On one hand, interventions need to be described in sufficient detail to enable surgeons to replicate them accurately in routine practice; however, too much standardisation may reduce the generalisability of results [6]. A balance between ‘adequate’ descriptions of interventions and the practicality of delivery is likely to be necessary [7].

The Medical Research Council guidance for developing and evaluating complex healthcare interventions (including surgery) suggests that centres participating in RCTs should ‘consistently provide as close to the same intervention as possible’ by ‘standardising the content and delivery of the intervention’ [3, 8]. It also recommends identifying and piloting the key components of interventions before full evaluation occurs. Whilst this may be helpful advice, it remains unclear how it should be implemented during the design of surgical RCTs. This study aimed to develop methods for identifying the key components of surgical interventions within RCTs in an ongoing randomised trial evaluating surgical procedures for patients with severe and complex morbid obesity (the By-Band study, HTA 09/127/53).

## Methods

Qualitative case study methodology was selected to enable the in-depth exploration of complex phenomena (surgical interventions) within their original setting (the operating theatre) in the context of the By-Band study [9, 10]. By-Band aims to compare the effectiveness and cost effectiveness of two surgical procedures for the treatment of patients with severe and complex obesity: laparoscopic adjustable gastric band (band) and laparoscopic Roux-en-Y gastric bypass (bypass) [11]. A gastric band aims to restrict the amount that patients can eat by placing a constricting ring (band) around the top of the stomach. The band incorporates an inflatable balloon to allow adjustment of the size of the stomach opening. Adjustments are undertaken by adding (inflating the balloon) or removing (deflating the balloon) saline through a subcutaneous access port. Placement of a band is considered to be a less invasive procedure than other types of bariatric surgery, conferring fewer technical challenges and lasting approximately 30 minutes [12]. Bypass aims to restrict the amount that patients can eat and prevent the complete absorption of food. This involves reducing the stomach to a small pouch (approximately the size of a thumb), and diverting the flow of food away from the remaining stomach and proximal small intestine. Current evidence suggests that bypass is a complex procedure, lasting between 45 minutes and 2 hours and requiring advanced technical skills [13, 14]. In view of this complexity, and the fact that the operative steps of bypass are less well established than band, bypass was selected for the case studies.

The By-Band study is an RCT with an internal pilot (phase 1), which was primarily designed to establish whether it was possible to recruit. The pilot phase provided an opportunity to use case studies to explore the surgical interventions and inform their description and standardisation in the main trial (phase 2). The study received full approval from the South West - Frenchay Research Ethics Committee (11/SW/0248). Consent for video recording operations was obtained from patients concurrently with authorisation for randomisation within the By-Band study. All surgeons and wider operating team members were asked if they were willing to be observed in theatre and interviewed.

## Centres and surgeons

The two phase 1 centres both offer specialised bariatric services and perform over 200 procedures every year. Both comprise a team of three consultant surgeons, of which one is not participating in the trial. Participating surgeons’ experience is described in Table 1.

**Table 1:** Summary of surgeons’ experience, by centre

Centre	Surgeon	Number of years as a consultant	Approximate number of bypass procedures performed
A^a^	S1	11	500+
	S3	1.5	130
	S4	N/A	50
	S5	7	500+
B^~^	S2	11	500+
	S6	7	400

*N/A* not applicable

^a^Although there are three consultant surgeons in Centre A, four surgeons were included in the case studies. S4 was a senior surgical trainee undertaking a bariatric fellowship

^~^Although there are three consultant surgeons in Centre B, one was not participating in the By-Band study

## Sampling strategy

Bypass procedures performed at both centres were purposively sampled as cases for the study. Case studies typically involve much smaller numbers of participants than other forms of research. Each case, however, is studied intensively and often contains multiple variables of interest and data sources, which yields a large amount of information [15]. Consequently, case studies may be particularly helpful when there is a need to investigate phenomena (or interventions) in great detail. Cases were chosen to encompass surgeons of varying experience from both study centres, up to the point where additional data were not adding anything new to the analytical framework and saturation was considered to have been achieved.

## Data collection

Each case study involved the collection of multiple data sources: digital video recordings of the procedure, non-participant observations and interviews with the operating surgeons. Digital video recordings were collected directly from the laparoscopic equipment already in routine use in the operating theatre. Recording started when the surgeon placed the camera into the abdomen and ended when it was removed after the procedure had finished. Concurrent non-participant observation of the operation was undertaken, to enable documentation of verbal and non-verbal communication and contextual factors. Observations were recorded by hand onto an observation schedule, which was developed during early visits to the operating theatre prior to the commencement of data collection (Appendix 1). All surgeons whose operations were video recorded and observed were invited to participate in two interviews. The first was undertaken immediately after the operation to discuss whether the procedure had progressed smoothly, and to identify and explore reasons behind any unusual events or deviations from the usual procedure. The second follow-up interview was undertaken after analysis of the case study data had commenced, often within 3 weeks of the initial surgery, to enable discussion of similarities and differences between centres and surgeons in terms of how the operation was performed. In addition, views about standardisation of the operation were explored. Interviews were semi-structured and directed by topic guides, which were developed based on existing literature and clinical knowledge (Appendices 2 and 3). Guides consisted of a list of open-ended questions to ensure that all topics were covered in each interview but were sufficiently flexible to enable issues of importance to emerge. The topic guides were adapted as interviews and analyses progressed, to explore emergent findings.

The case studies were undertaken by a medically qualified surgical trainee (NB) with no direct clinical experience of the procedures under investigation. She had not previously worked in either of the hospitals participating in the By-Band study, and was unfamiliar with the surgeons, teams, and operating theatre environments.

## Data analysis

Data collection and analysis ran in parallel. NVivo 10 (QSR International, Melbourne, Australia) was used to aid the storage and analyses of all types of data.

### Digital videos and non-participant observations

The digital videos of surgical procedures were viewed unedited, and transcribed from beginning to end. The process involved the researcher watching and re-watching the recording in order to document movements, instruments and actions that were visible on the screen. Movements and actions from the video transcript were grouped together into operative steps, which generated a stepwise account of the procedure. Notes from the observations were written up as soon as possible afterwards. These two types of data were synchronised for each case. Synchronisation involved alignment of each non-participant observation with the respective operative step(s) from the video recording. The synchronised data were studied in depth, enabling steps to be compared and contrasted between surgeons and centres. This iterative process generated findings relating to the (un)usual steps and crucial aspects of the operation. Findings were subsequently tested and refined as further data were analysed, and explored in the interviews with surgeons.

### Post-operative and follow-up interviews

Interview data was used to confirm, challenge and clarify findings from the videos and observations. For example, if an unexpected event or step was identified within the video or observation data, this was explored with the surgeon in the post-operative interview. Interviews were transcribed verbatim. An inductive approach to data analysis was undertaken, enabling themes to be derived from the data [16]. Transcripts were coded by ascribing key words or phrases that captured the meaning of the text to identify common emerging themes. The coding index was added to, and coded material regrouped, as new themes and categories emerged from subsequent interviews. Further analysis involved scrutinising the textual data for differences and similarities within themes, and relating findings back to the video and observational analyses.

## Results

Eight case studies were undertaken, four in each centre. Video recordings varied in length from 45 to 142 minutes. For technical reasons, two procedures were not captured on video but extensive notes were made during the observations. Post-operative interviews with surgeons lasted between 5 and 15 minutes and were conducted face to face (n = 6) immediately after the procedure, or by telephone (n = 2) the following day. Follow-up interviews were conducted face to face within 8 weeks of the operation and lasted between 20 and 45 minutes. Findings relating to the constituents steps and crucial aspects, and surgeons’ views about standardisation of bypass procedures, are considered below.

### Identifying the constituent steps of bypass procedures

**Fig. 1:**
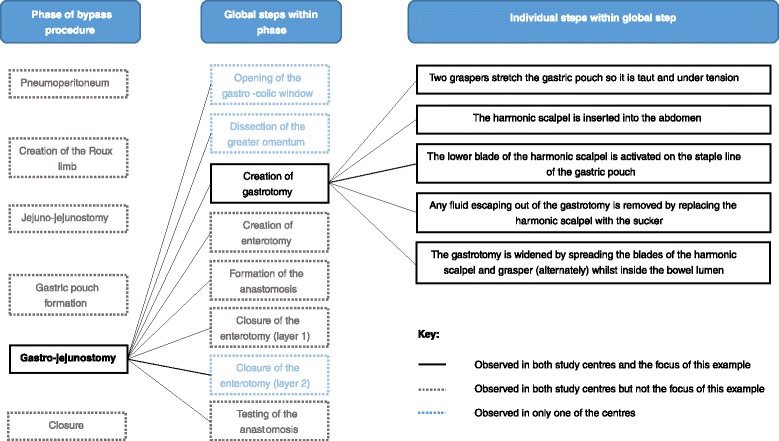
Identification of the steps within each phase of bypass, using gastro-jejunostomy as an example

The bypass procedure was split into six phases according to their purpose (Table 2). Although all six phases occurred in every case, the order in which they were completed varied between centres. Detailed analysis of the operative videos enabled stepwise accounts of the ‘usual steps’ of bypass procedures to be generated. Within each phase, similar steps were grouped into global steps, as shown in Fig. 1. Subsequently, similarities and differences in the way that surgeons performed global steps within each phase were established. These were used during surgeon interviews to understand the ‘crucial’ aspects of bypass and the potential extent of standardisation.

**Table 2:** The main phases of bypass procedures, by centre

Phase	Lay description of the purpose of each phase	Order phase performed	
		Centre A	Centre B
Establishing a pneumoperitoneum and insertion of ports	To provide access to the abdominal cavity	1	1
Creation of the Roux limb	To divert the passage of food away from the first portion of small bowel	2	3
Jejuno-jejunostomy		3	5
Gastric pouch formation	To reduce the stomach volume	4	2
Gastro-jejunostomy formation	To join the stomach pouch to the end of the diverted portion of bowel	5	4
Closure	To reconstruct the layers of fat and muscle, re-creating how they looked before the operation	6	6

### Identifying the crucial aspects of bypass

All surgeons mentioned specific aspects of bypass that they considered to be important. These included formation of the gastric pouch, the route of the Roux limb, closure of mesenteric defects and testing of the gastrojejunal anastomosis. These ‘crucial aspects’ were compared with findings from the digital video recordings and observations and together with the rationale provided by surgeons, are summarised in Table 3.

**Table 3:** Surgeons’ views about the crucial aspects of bypass

Operative phase	Observation and video findings	Surgeons’ rationale
Gastric pouch formation	All surgeons create a short, thin gastric pouch	*‘I think that the most important element is pouch size…I think probably from a hormonal element and also to, um, increase the speed of transit into the small bowel.’ [S3]*
	Surgeons in Centre A routinely use a bougie to help size the gastric pouch, whereas surgeons in Centre B do not	*‘I, I think the size of the pouch is probably, probably crucial, or at least I know a really small pouch is associated with a good 15-year weight loss.’ [S1]*
		*‘It depends what you mean by a bigger pouch. Are we, are we talking a difference of a centimetre or a difference of 20 centimetres? I think if you do…a horizontal pouch perhaps the way it was done years ago then that might make a difference. But if you do a vertical pouch, um, I don’t think it matters if it’s 6 centimetres or 10 centimetres or probably even 15 centimetres, as long as it’s a narrow pouch and you’ve excluded the cardia. I know the chaps from [name of country] make long pouches and…they seem to have good results.’ [S5]*
		*‘I don’t think it is [important]. I mean, it’s done for a number of reasons. You can do it to make sure that your pouch is not too wide. I think more people do it to make sure that they don’t accidentally make the anastomosis too small…But it does…it makes you feel happier.’ [S5]*
Roux limb formation	Surgeons in Centre A use the retrocolic route	*‘…allows you to make a smaller gastric pouch. If you’re doing an antecolic anastomosis you, you must do a much, probably much longer gastric pouch, um, halfway towards a gastric sleeve almost ….So if you start off with a big pouch you have to think well, the likelihood of it actually getting enlarged over this time is, is going to be there…’ [S1]*
	Surgeons in Centre B use the antecolic route	*‘…if you go antecolic you don’t get the problem with the mesocolic window…occasionally you have to take a patient back because it’s too tight, um, as you go in the retrocolic tunnel, and it’s one of the more common places for a hernia, in the mesocolic window.’ [S6]*
Closure of mesenteric defects	Surgeons in Centre A routinely close the defects	*‘Personally I think it’s crazy not to close them…I don’t think you can be too prescriptive because it is a pragmatic trial. Closing an internal defect is preferable but if you don’t do it, it doesn’t mean you can’t be in the study.’ [S1]*
	Surgeons in Centre B do not close the defects	*‘…the consensus was that medico-legally, regardless of the evidence, it was indefensible not to close the defects.’ [S2]*
Testing of the gastrojejunal anastomosis	Performed by all surgeons in Centres A and B	*‘Um why – you sleep, it’s probably good for the surgeon. I think, um, yes some sort of leak test is probably a good idea, yes.’ [S3]*
		*‘…touch wood, I haven’t had a [positive] blue dye test for about 5 years. So is it important? Probably not.’ [S5]*

### Surgeons’ views about standardisation of bypass

Initially, surgeons held mixed views on whether bypass should be standardised within the By-Band study. One of the surgeons expressed a clear preference that surgery should be highly standardised, linking this to the fact that the trial could be criticised if the operations were not performed in the same way:

S6: *‘In the overall game you’re trying to, you’re trying to be exact with the surgery. You have to, it’s [standardisation] the only way to do it, cos otherwise you’ll just get torn down and criticised for not doing it.’*

Conversely, other surgeons did not consider that standardisation was important:

S2: *‘Um, I just don’t think it’s of huge importance…as long as the operation is done safely, um, I really don’t think it matters. It’s like whether you brush your hair forwards or backwards or the side parting is on the left or the right. I really don’t think it matters.’*

Surgeons’ views about the standardisation of bypass focused on a lack of available evidence, the need to be pragmatic and achieving ‘buy-in’ from other surgeons. These are detailed below.

#### (Lack of) available evidence

All of the surgeons expressed opinions about the way in which bypass should be performed. Despite this, they conceded that there was a general lack of evidence that particular operative techniques conferred better outcomes. One surgeon specifically mentioned this in relation to weight loss, which is considered to be one of the main objectives of bariatric surgery:

S1: *‘The, the problem is a lot of what we’re saying is just, it’s just, it’s not evidence-based. There isn’t any evidence…my interpretation of the literature is that the weight loss appears to be the same, um, irrespective of which technique you used.’*

The surgeons stated that it would be difficult to decide on how standardisation of bypass should be achieved, because of the lack of evidence of its effectiveness:

S5: *‘If there’s still big arguments about it how do you standardise it? So how can you standardise when there’s no consensus?’*

#### The need to be pragmatic

Most surgeons felt that strict standardisation of bypass would prevent the By-Band study from reflecting the entire spectrum of bariatric practice:

S5: *‘We know that worldwide people do it different ways. It’s, it’s probably good that you’ve got both techniques as part of the study to be honest…If you want to know the difference between gastric bypass and gastric band in the UK you need to include all comers. Um, and the more of a spread of everything you get the better.’*

The need for pragmatism was balanced with the potentially detrimental impact of strict standardisation:

S2: *‘I think you have to have an open mind, you have to be pragmatic because at the end of the day it’s supposed to be a pragmatic study. It’s not a study designed to compare one bypass with another with another…if you overly complicate it and become too non-pragmatic then people will not comply.’*

In balancing pragmatism with rigidity, surgeons described a compromise in the form of ‘ground rules’ about how the operation should be performed within the trial:

S2: *‘I think there should be some ground rules. I think that what we’ve got at the moment is pragmatic and I think we should try to be doing something that’s similar and…it needs to not be grossly dissimilar…I think we want to be, we want to be pragmatic but we want to be reasonably clear that what we’re doing is representative.’*

#### Achieving ‘buy-in’ amongst surgeons

All surgeons agreed that strict standardisation of bypass procedures might influence whether future surgeons would consider participating in the By-Band study:

S6: *‘You can standardise it and what’ll happen is you will…certain surgeons will exclude themselves from the, from the operation cos they won’t agree with it for whatever…If we mandate retrocolic, you’ll get a lot of people not wanting to bother, not wanting to go into the trial.’*

They related this to the fact that surgeons often had fixed views about the procedure and would not want to perform it in a different way:

S4: *‘You can standardise to an extent, but you can’t impose it onto surgeons because surgeons won’t be imposed onto as I am sure you are aware… Different surgeons will do what they want to do.’*

## Discussion

This novel exploratory work used qualitative case studies to identify the key components of surgical interventions within an RCT. The case studies used a combination of digital video recordings, non-participant observation and interviews with surgeons to explore bypass procedures in detail. This provided important insights into how a relatively common surgical procedure is delivered differently by different surgeons and centres. Identification of these differences informed discussions about the crucial aspects of bypass. Although surgeons agreed that the most critical component was the size and shape of the gastric pouch, there was no consensus regarding other aspects of the procedure. They conceded that evidence about the ‘best way’ to perform bypass was lacking and, combined with the pragmatic nature of the By-Band study agreed that strict standardisation of bypass might not be required. However, they considered that some ‘minimum standards’ might be important in ensuring that procedures were performed in broadly similar ways. This information has shaped the design of the intervention in phase 2 of By-Band and consequently, the data collection of key components of bypass during the trial.

Well designed and conducted RCTs provide robust evidence to determine the effectiveness of interventions, allowing clinicians to make evidence-based treatment decisions. Reporting of RCT design and delivery is also necessary to facilitate transparency, critical appraisal and systematic reviews, and to enable results to be implemented in practice. The Consolidated Standards of Reporting Trials (CONSORT) statement provides a checklist of reporting standards for RCTs [17], with a specific extension for non-pharmacological treatments (CONSORT-NPT) [18], including surgery. CONSORT-NPT recommends that ‘precise details of the experimental treatment and comparator’ are provided, together with ‘details of how the interventions were standardised’. Despite this guidance, however, reporting standards have remained poor. A recent systematic review of 80 trials, reporting details of 160 surgical interventions, found that at least some description (other than simply the name of the operation) was provided for 129 (81 %) of the 160 interventions and 47 (29 %) were reported to be standardised in some way [19]. Of these 47, 30 (64 %) were accompanied by a statement that the intervention had been standardised, although no information was provided about how this was achieved.

However, it may not always be necessary nor appropriate to standardise each component or step of surgical interventions. This should be driven by the research question, the interventions being compared and the approach to trial design [20]. In explanatory trials, which determine the efficacy of interventions, great detail may be necessary because the interventions are often novel and their safety assessed within carefully controlled settings. Pragmatic designs, which determine whether interventions are effective in the real world, are often multi-centred with large numbers of surgeons. Under such circumstances, specifying each operative step is likely to create difficulties, and ensuring that each step was delivered as planned may be unrealistic. A balance between ‘adequate’ standardisation and practicality is therefore necessary and appropriate. One potential approach would be to standardise an intervention’s key ‘functions’ rather than specifying the specific ‘form’ it needs to take [21]. This assumes, however, that a surgical intervention represents the creation of a new anatomical situation irrespective of the individual steps taken to create it. A compromise would be to determine the minimum active ingredients of the intervention – i.e. those that are thought to optimally influence outcomes or those which are different between the interventions in each trial group – and the degree to which they need to be standardised. The current study has gone some way to developing methods for understanding how surgical interventions are standardised in explanatory and pragmatic trials. Applying these methods more widely may help to identify key components of interventions that can be agreed upon and delivered in trial centres, which in turn allows monitoring of those components during the trial and reporting adherence to the intervention protocol possible. This will improve the transparency of reporting of surgical interventions within RCTs in accordance with existing guidelines and allow results of trials with a positive treatment effect to be subsequently implemented in practice.

To our knowledge, this is the first study to undertake an in-depth exploration of surgical interventions in RCTs. It can be difficult to separate complex interventions (such as surgery) from the context in which they are delivered. Conducting case studies in the operating theatre may therefore enable researchers to gather a ‘true’ picture of surgical interventions within the environment in which they are delivered, offering insights that might not have been achieved using other approaches [15]. Another advantage of collecting ‘naturalistic’ empirical data (in the form of observation and videos) is the opportunity for triangulation with ‘generated’ data from surgeon interviews, enriching and improving the validity of the study findings. Undertaking case studies may, therefore, facilitate an understanding of how interventions are actually performed (thereby disentangling the differences between what surgeons say they do and what they actually do), explore variations in intervention delivery and understand views about standardisation of interventions in the context of trials. Despite these advantages, there are some limitations relating to the challenges associated with the impact of the researcher. The case studies were undertaken by a trainee surgeon meaning that existing knowledge and pre-conceptions may have influenced the content and interpretation of the data. In contrast to the researcher, all of the surgeons in the case studies were male with many years’ experience of bariatric surgery. This imbalance may have been beneficial because the researcher did not have pre-existing ideas or expectations about how the operations should be performed. Alternatively it may have introduced pre-conceptions that would not have occurred if a non-clinical researcher had conducted the case studies.

Undertaking case studies may facilitate an understanding of how interventions are actually performed, and understand views about standardisation of interventions in the context of trials. By establishing the key components of interventions and variations in their delivery, case studies may help to explore the effect of individual surgeons and centres (which can lead to performance bias) by understanding and quantifying the main similarities and differences between surgeons and centres. This has the potential to inform the way in which stratification processes and multivariate analyses (to account for performance bias) are undertaken. However, investigating the entire intervention in this way may be time consuming and generate large volumes of data. One solution might be to consider the constituent components and steps of surgical interventions, and how they could be standardised, before undertaking case study work. The case studies could then be targeted towards areas of uncertainty (for example, any component that surgeons are unable to agree on). To this end, a typology has been developed that provides guidance for surgeons and trialists to use during trial design [22]. It aims to provide a structure for ascertaining the constituent components and steps of interventions, and for considering how standardisation may be achieved during trial delivery. One problem with this approach in isolation is that although individual surgeons may know the key components of a surgical procedure, opinions may differ between surgeons. Different surgeons operate in different ways meaning that the way in which surgical interventions are delivered – between and within centres, regions, countries and continents – is variable. When used in conjunction with the case study work, the typology can identify the key components of an intervention, including differences in the ways they are delivered. Moreover, the boundaries within which they should subsequently be delivered within an RCT can be agreed (by surgeons involved in the trial and ideally, the wider surgical community). As such, surgeons reading trial protocols and final reports can understand what operations were performed and how, increasing the likelihood that effective interventions are implemented as intended in practice, reducing criticisms such as ‘the result would have been different if the operation was done my way’.

## Conclusions

Randomised controlled trials in surgery can be difficult to design and conduct, and one of the reasons for this is that surgical interventions are complex. Case studies provide a way of exploring this complexity to make sense of how surgical interventions are described and standardised in trials. Successful completion of case studies within the operating theatre has developed methods that can be transferred to intervention design and delivery within the broader context of surgical RCTs. They enable the components and steps of interventions to be established, including those which are considered as crucial, and the extent to which standardisation may be required. This has the potential to influence the way in which surgical interventions are designed and delivered within RCTs. It is now necessary to test the application of this case study methodology in other settings to evaluate its usability, usefulness and acceptability.

## Abbreviations

Band: Laparoscopic adjustable gastric band
Bypass: Laparoscopic Roux-en-Y gastric bypass
CONSORT-NPT: Consolidated Standards of Reporting Trials for Non-Pharmacologic Treatment
RCT: Randomised controlled trial
